# Antibiotic Resistance Profile and Bio-Control of Multidrug-Resistant *Escherichia coli* Isolated from Raw Milk in Vietnam Using Bacteriophages

**DOI:** 10.3390/pathogens13060494

**Published:** 2024-06-09

**Authors:** Hoang Minh Duc, Tran Thi Khanh Hoa, Cam Thi Thu Ha, Le Van Hung, Nguyen Van Thang, Hoang Minh Son, Gary A. Flory

**Affiliations:** 1 Department of Veterinary Public Health, Faculty of Veterinary Medicine, Vietnam National University of Agriculture Trau Quy, Gia Lam, Hanoi 12400, Vietnam; 2 Veterinary Hospital, Faculty of Veterinary Medicine, Vietnam National University of Agriculture Trau Quy, Gia Lam, Hanoi 12400, Vietnam; 3 Department of Anatomy and Histology, Faculty of Veterinary Medicine, Vietnam National University of Agriculture Trau Quy, Gia Lam, Hanoi 12400, Vietnam; 4 G.A. Flory Consulting, Mt. Crawford, VA 22841, USA

**Keywords:** *E. coli*, bacteriophages, antibiotic resistance, multidrug resistance

## Abstract

*E. coli* is an important zoonotic pathogen capable of causing foodborne illness and bovine mastitis. Bacteriophages have been increasingly considered a promising tool to control unwanted bacteria. The aim of this study is to determine the antibiotic resistance profile of *E. coli* isolated from raw milk and the efficacy of phage in controlling multidrug-resistant *E. coli* in raw milk. Antibiotic susceptibility testing showed the highest resistance rates of *E. coli* isolates to co-trime (27.34%) and ampicillin (27.34%), followed by streptomycin (25.18%), tetracycline (23.02%), and the lowest resistance rates to ciprofloxacin, gentamycin, and ceftazidime, all at a rate of 2.16%. All isolates were susceptible to meropenem. Of the 139 *E. coli* isolates, 57 (41.01%) were resistant to at least one antibiotic, and 35 (25.18%) were classified as MDR strains. Molecular characterization indicated that 5 (3.6%) out of the 139 isolates were STEC strains carrying *stx1* gene. Seven (5.04%) isolates were phenotypically identified as ESBLEC, and four isolates (2.88%) were resistant to colistin. The results of the genotypic test revealed that four out of seven ESBLEC strains carried both *bla_TEM_* and *bla*_CTX-M-1_, two harbored *bla_TEM_*, and one possessed *bla*_CTX-M-1_, while *mcr-1* was detected in all four colistin-resistant *E. coli* isolates. In particular, one isolated *E. coli* strain (EM148) was determined to be a multidrug-resistant strain simultaneously carrying *bla_TEM_*, *bla*_CTX-M-1_, and *mcr-1*. A total of eight phages were successfully recovered from raw milk. The application of phage PEM3 significantly reduced viable counts of multidrug-resistant host EM148 in raw milk by at least 2.31 log CFU/mL at both 24 °C and 4 °C.

## 1. Introduction

Food-borne diseases pose a major public health concern worldwide [1] and cause 2 million deaths annually in developing countries [2]. *Escherichia coli* is a Gram-negative bacterium that colonizes the lower intestine of humans and animals [3]. Therefore, *E. coli* has been employed as a bacterial indicator of fecal contamination to evaluate the hygiene of foodstuffs for almost a century [3]. Additionally, *E. coli*, particularly Shiga-toxin-producing *E. coli* (STEC), has been known to be one of the most important foodborne pathogens due to their toxicity and infectious potential [4]. STEC can cause food poisoning even at very low doses and produce cytotoxins called Shiga toxin 1 (Stx1) and Shiga toxin 2 (Stx2), leading to hemorrhagic colitis, thrombotic thrombocytopenic purpura, and hemolytic uremic syndrome (HUS) [4]. *E. coli* has been also recognized as the most commonly used indicator of the selective pressure imposed by antimicrobial use in livestock [5], as they can acquire and transfer resistant genes to bacterial strains in the same genus and even in different genera, including commensal and pathogenic bacteria in the intestinal tract, which may pose a risk to human and animal health [6]. In addition, *E. coli* is recommended for antimicrobial resistance (AMR) monitoring in livestock, humans, and the environment, as the bacteria can be easily and inexpensively isolated and preserved [7]. Multidrug-resistant *E. coli*, particularly extended-spectrum *β-*lactamases-producing *E. coli* (ESBLEC), have been classified as the most urgent health issue by the World Health Organization (WHO) [8]. Infection with ESBLEC results in a limitation of therapeutic options. Colistin has been considered to be one of the last-resort drugs for the treatment of ESBLEC infections. However, it has been recently reported that ESBLEC resistant to colistin was present in raw milk in China [9].

Nowadays, the demand for raw milk is increasing worldwide [10,11]. In fact, raw milk has recently been approved for human consumption in 30 states in the US [12,13] and approximately 3% of the US population consumes it [14]. Although raw milk can provide several health benefits to humans, the consumption of raw milk may cause foodborne illnesses as raw milk has been reported to contain various foodborne pathogens (STEC, *Salmonella*, and *Staphylococcus aureus*), and even antibiotic-resistant microbes, including multidrug-resistant *E. coli* [15,16].

The exploitation of natural antimicrobials to control foodborne pathogens has increasingly gained attention due to the growing customer demand for healthy, nutritious, and minimally processed food products without chemical preservatives [17]. Bacteriophages are one of the most interesting natural food additives approved by the Food and Drug Administration (FDA) [18,19]. Bacteriophages are defined as bacterial viruses only infecting specific bacteria. Therefore, they do not have the mechanism to infect human and animal cells, and do not disturb beneficial microorganisms present in food and the intestinal tract [20,21]. Additionally, phages are unlikely to affect the organoleptic properties of foods [22]. Moreover, phages can destroy antibiotic-resistant bacteria [23,24]. Due to these advantages, a variety of studies have focused on the use of phages to control foodborne pathogens [22,25,26].

The prevalence, antibiotic resistance, and biocontrol of *E. coli* in raw milk have been reported in various countries [27,28,29,30,31,32,33]. However, there is limited information about the prevalence, antibiotic resistance, and bio-control of multidrug-resistant *E. coli* isolated from raw milk in Vietnam. Therefore, the aim of this study was to investigate the prevalence, antibiotic resistance, and bio-control of multidrug-resistant *E. coli* isolated from raw milk in Vietnam using lytic phages.

## 2. Materials and Methods

### 2.1. Isolation and Identification of E. coli

A total of 400 raw milk samples were collected from raw milk markets in Ba Vi District (*n* = 200) and Phu Dong, Gia Lam District, Hanoi (*n* = 200). The collected samples were kept in ice boxes and brought back to the laboratory for *E. coli* detection and isolation within 24 h. Briefly, raw milk samples (25 mL) were homogenized with 225 mL of buffered peptone water (BPW, Oxoid, ThermoFisher, Hants, UK) and incubated at 37 °C for 24 h. The samples were then streaked onto MacConkey agar (Oxoid, ThermoFisher, Hants, UK) and incubated at 37 °C for 24 h. Following incubation, typical *E. coli* colonies (pink, round, smooth, non-mucoid) on MacConkey agar were collected and re-cultured on Eosin Methylene Blue agar (EMB, Oxoid, ThermoFisher, Hants, UK). *E. coli* colonies on EMB agar (black center and green sheen) were selected for biochemical identification using the API-20E kit (bioMérieux, Marcy I’Etoile, France) following the instructions of the manufacturer. Biochemically identified *E. coli* isolates were further confirmed by PCR according to a previously described method [34].

### 2.2. Antibiotic Susceptibility Test

The antibiotic susceptibility of the *E. coli* isolates was determined using the agar dilution method according to the standards and interpretation criteria described by the Clinical and Laboratory Standards Institute (CLSI, 2018). A total of 15 different antimicrobials were applied, including ampicillin, tetracycline, streptomycin, gentamicin, colistin, azithromycin, trimethoprim/sulfamethoxazole, florphenicol, meropenem, cefotaxime, cefoxitin, cefepime, ceftazidime, ciprofloxacin, and nalidixic acid. *E. coli* strain ATCC 25922 was used as a quality control strain organism. Isolates resistant to 3 or more antibiotic classes were regarded as MDR strains. *E. coli* isolates resistant to cefotaxime and/or ceftazidime were confirmed to be capable of phenotypical ESBL production by a synergy test using cefotaxime and ceftazidime with and without clavulanic acid (Oxoid, ThermoFisher, Hants, UK) according to the CLSI guidelines (CLSI, 2018). 

### 2.3. Detection of STEC Virulence-Associated Genes, ESBLs Encoding Genes, and mcr Genes

The DNA of the *E. coli* isolates was extracted by the GeneJet Genomic DNA purification kit (Thermoscientific, Vilnius, Lithuania). Isolated *E. coli* strains were tested for the presence of STEC virulence-coding genes by multiplex PCR [35,36]. The primers used for PCR in this study are detailed in Table 1. The thermal cycle of PCR reaction was as follows: denaturation at 94 °C/5 min; 35 cycles of denaturation at 94 °C/30 s, annealing at 67 °C/80 s, and extension at 72 °C/90 s; and a final step was 72 °C/10 min.

The phenotypically ESBL-identified isolates were tested for the presence of *β*-lactamase encoding genes using the multiplex PCR following a previously described method with some modifications [37]. The primers used for PCR in this study are listed in Table 2. The PCR was performed under the following conditions: a denaturation phase at 95 °C/5 min; 25 cycles with a denaturation phase at 95 °C/30 s, annealing at 60 °C/90 s, and extension at 72 °C/90 s, followed by a final extension at 68 °C for 10 min.

All colistin-resistant isolates were screened for *mcr* genes, including *mcr-1*, *mcr-2*, *mcr-3*, *mcr-4*, and *mcr-5*, using multiplex PCR according to the previous method described by [38]. The primers used for the detection of *mcr* genes are shown in Table 3.

### 2.4. Phage Isolation, Purification, and Propagation 

ESBLEC (EM148) co-carrying *bla_TEM_*, *bla*_CTX-M-1_, and *mcr-1* genes was selected as a bacterial host for phage isolation. A total of 50 raw milk samples were purchased from local markets in Hanoi for the isolation of phages against *E. coli* EM148. Phage isolation, purification, and propagation were performed according to the previously described protocol [22]. Briefly, 10 mL of raw milk was inoculated with 100 μL of overnight culture of EM148, and incubated overnight at 37 °C with shaking. After incubation, the sample was centrifuged at 12,000× *g* for 5 min at 4 °C. The supernatant was collected and filtered through a 0.45 μm pore size membrane filter (Merck Millipore, County Cork, Ireland) to produce phage lysate. The lysate was serially diluted in saline magnesium (SM) buffer (0.05 M Tris-HCl buffer, pH 7.5, containing 0.1 M NaCl, 0.008 M MgSO_4_, and 0.01% gelatin), mixed with 100 μL of overnight EM148 culture and 4 mL of molten top agar (Luria Bertani (LB, (TSA; Becton, Dickinson and Company, Franklin Lakes, NJ, USA) broth with 0.4% (*w*/*v*) agar). The mixture was poured on TSA and incubated overnight at 37 °C. A well-separated plaque was collected and placed in SM buffer. The plaque suspension was serially diluted in SM buffer again, mixed with EM148 culture and molten top agar, and then poured on tryptic soy agar (TSA; Becton, Dickinson and Company, NJ, USA) for phage purification. Following incubation, a single plaque was collected for further cycles. This purification process was repeated for at least three cycles. The purified phage was propagated to obtain a high titer before being stored at 4 °C for further experiments.

### 2.5. Phage Characterization

The host range of isolated phages was determined on 139 *E. coli* isolates by dropping 10 μL of phage suspension on the surface of the double-layer agar inoculated with the bacterial host. The plates were then incubated overnight at 37 °C for the formation of plaque. Clear plaque was regarded as the phage lysis. On the contrary, the absence of clear plaque indicated no lysis.

The one-step growth curve of phage PEM3 in bacterial host EM148 was determined according to a previously described method [40]. Briefly, EM148 was cultured in 5 mL of LB broth to reach a cell concentration of around 10^8^ CFU/mL. The bacterial culture (1 mL) was then mixed with 1 mL of phage suspension to obtain the multiplicity of infection (MOI) of 0.01. The mixture was incubated for 10 min at 37 °C to allow the phage particles to attach to the host cells. Afterward, the mixture was centrifuged at 10,000× *g* for 30 s at room temperature to remove the supernatant containing unadsorbed phages. The pellet was resuspended in 10 mL of fresh LB broth and placed in a water bath with shaking at 37 °C. Samples (100 μL) were collected at 5 min intervals and subjected to a double-layer agar assay to determine the phage titers. 

The stability of phage PEM3 was investigated under various temperatures, pH levels, and NaCl conditions. For thermal stability, phage suspension (100 µL; 5 × 10^10^ PFU/mL) was mixed with 5 mL of SM buffer to obtain the phage titer of 10^9^ PFU/mL before incubating it in a water bath with shaking at a wide range of temperatures (40–90 °C) for 30 min. To determine the pH stability, 100 μL of phage suspension (5 × 10^10^ PFU/mL) was inoculated into SM buffer (5 mL), preadjusted to various pH values (2–13), and then incubated at 37 °C for 60 min. For NaCl tolerance, phage suspension (5 × 10^10^ PFU/mL) was added to 5 mL of NaCl solutions at different concentrations (1–11%) and kept in a shaking water bath at 37 °C for 60 min. The phage titer was determined after the heat, pH, and NaCl treatments by the double-layer agar method, as mentioned above.

### 2.6. Efficacy of Phage PEM3 against EM148 in LB Broth and Raw Milk

To evaluate the effectiveness of phage PEM3 against *E. coli* EM148 in vitro, 100 µL of the bacterial culture (5 × 10^6^ CFU/mL) was inoculated into 5 mL of LB broth and treated with 100 µL of phage suspension (5 × 10^9^ PFU/mL). For a control, SM buffer (100 µL) was used instead of phage suspension. The mixture was then incubated at 24 °C and 4 °C. At 2, 4, 6, and 24 h after incubation, 100 μL of the mixture was withdrawn and serially diluted in phosphate-buffered saline (PBS; 137-mM NaCl, 8.10-mM Na_2_HPO_4_, 2.68-mM KCl, 1.47-mM KH_2_PO_4_). Appropriate dilutions (100 µL) were plated on TSA agar and incubated at 37 °C for 24 h. Viable counts of EM148 were calculated the next day.

The efficacy of phage PEM3 in reducing viable counts of *E. coli* EM148 was also determined in raw milk. Raw milk (5 mL) was artificially spiked with 100 µL of EM148 (5 × 10^6^ CFU/mL), treated with 100 µL of phage PEM3 (5 × 10^9^ PFU/mL), and then stored at 24 °C and 4 °C for 24 h. The control was inoculated with SM buffer instead of phage suspension. At 2, 4, 6, and 24 h after incubation, the sample (100 µL) was collected and appropriately diluted in PBS before plating on MacConkey agar supplemented with cefotaxime (4 mg/L) and colistin (4 mg/L) and incubated at 37 °C for 24 h. After incubation, the viable counts of EM148 were enumerated. The experiments were repeated at least three times. The viable counts were calculated as the mean values and standard deviations of the means. The significance of the differences between the treatments and controls was determined by a *t*-test (Microsoft Excel 2016 for Mac OS).

## 3. Results

### 3.1. Prevalence of E. coli in Raw Milk

Among the 400 raw milk samples tested, 139 (34.75%) were positive for *E. coli.* To avoid duplication, only 139 *E. coli* strains were isolated and preserved from the positive samples (one isolate from each positive sample) at −70 °C for further use. This included 61 strains from the Ba Vi samples and 78 strains from the Gia Lam samples. The findings also revealed that the prevalence of *E. coli* in the raw milk samples collected from Gia lam (39%) was higher than in those from Ba Vi (30.5%).

### 3.2. Antimicrobial Susceptibility Profile

The results of the antimicrobial susceptibility test show that *E. coli* isolates exhibited the highest resistance rates to co-trime (27.34%) and ampicillin (27.34%), followed by streptomycin (25.18%) and tetracycline (23.02%) (Table 4). On the contrary, the resistance rates to ciprofloxacin (2.16%), gentamycin (2.16%), and ceftazidime (2.16%) were the lowest. Resistance to meropenem was not recorded in this study.

The isolates from Ba Vi showed the highest resistance rates to co-trime (44.26%), streptomycin (36.07%), ampicillin (36.07%), and tetracycline (34.43%) (Table 4). In contrast, the lowest resistance rates were observed with cefoxitin (1.64%), ceftazidime (3.28%), gentamycin (3.28%), ciprofloxacin (3.28%), and colistin (3.28%). The isolates from Gia Lam were highly resistant to ampicillin (20.51%), streptomycin (16.67%), tetracycline (14.10%), and co-trime (14.10%) (Table 4). On the other hand, the isolates showed the lowest resistance rates to ceftazidime (1.28%) and ciprofloxacin (1.28%). All the isolates from Ba Vi and Gia Lam were susceptible to meropenem.

A total of seven *E. coli* isolates were found to be resistant to ceftotaxime and/or ceftazidime (Table 4). These isolates were selected for the detection of ESBL phenotype. The antimicrobial susceptibility test also revealed that four *E. coli* isolates were resistant to colistin, and these isolates were used for the detection of colistin resistance genes (Table 4).

The results in Table 5 reveal that 41.01% (57/139) of the *E. coli* isolates were resistant to at least one antibiotic, and 25.18% (35/139) were determined as MDR. The MDR rate in raw milk collected from Ba Vi (34.43%) was higher than Gia lam (17.95%). A total of 27 antibiotic resistance patterns were identified (Table 5). The most common resistance pattern observed in the MDR isolates was AMP-STR-TET-SXT (nine isolates; 6.47%), followed by AMP-STR-TET-FLO-SXT (six isolates; 4.32%), and AMP-STR-TET-AZM-SXT (four isolates; 2.88%) (Table 5).

### 3.3. Detection of STEC Virulence-Associated Genes, ESBL Phenotype, β-Lactamase Encoding Genes, and mcr Genes

The results of the multiplex PCR revealed that 5 (3.6%) out of 139 *E. coli* isolates were positive for *stx1* gene. The *stx2* gene was not detected in any isolates. Among the five isolated STEC strains, one belonged to serogroup O157, while the remaining four isolates were unserotypeable. The *E. coli* O157 isolate simultaneously carried the *stx1*, *eae*, and *ehxA* genes. Three out of four of the non-O157 STEC isolates harbored both *stx1* and *ehxA*; the rest possessed *stx1* and *eae* genes.

The synergy test showed that 7 out of 7 (100%) cefotaxime-resistant isolates were identified as ESBL producers, of which 5 and 2 strains were isolated from Ba Vi and Gia Lam, respectively. This indicates that 5.04% (7/139) of the *E. coli* isolates could produce ESBL, and 1.75% (7/400) of the raw milk samples were positive for ESBLEC. These seven ESBL isolates were resistant to five or more antibiotics. The multiplex PCR analysis revealed that all seven ESBL-producing isolates carried at least one *β*-lactamase encoding gene, of which four isolates harbored two genes (*bla_TEM_*, *bla*_CTX-M-1_), two isolates carried only *bla_TEM_*, and one isolate harbored only *bla*_CTX-M-1_ (Table 6). The most frequently detected *β*-lactamase encoding genes were *bla_TEM_* (6/7; 85.71%), followed by *bla*_CTX-M-1_ (5/7; 71.42%). On the other hand, *bla*_SHV_, *bla*_CTX-M-2_, *bla*_CTX-M-8/25_, and *bla*_CTX-M-9_ were not detected in any ESBL-producing isolates tested. The colistin resistance gene (*mcr-1*) was present in all four (100%) colistin-resistant isolates. One *E. coli* strain (EM148) was found to carry both *β*-lactamase encoding genes (*bla_TEM_*, *bla*_CTX-M-1_) and the colistin resistance gene (*mcr-1*).

### 3.4. Phage Isolation and Characterization

**Figure 1:**
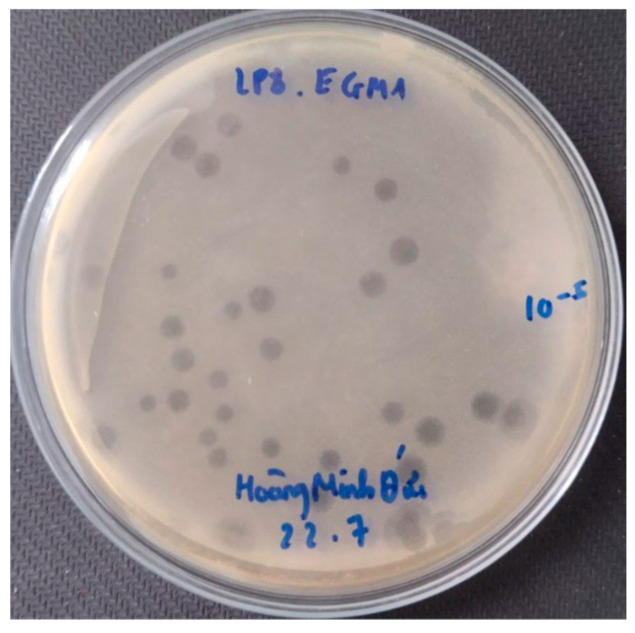
The clear plaques of phage PEM3.

A total of eight phages were isolated using *E. coli* EM148 as a bacterial host in this study. Of the eight isolated phages, six phages (PEM1, PEM2, PEM3, PEM5, PEM6, and PEM8) capable of forming clear plaques and generating stocks of high titer (>10^9^ PFU/mL) were selected for characterization (Figure 1).

The results of the host range show that phage PEM3 had the widest lytic spectrum, lysing 88 (63.3%) of the 139 *E. coli* isolates tested. On the contrary, phage PEM5 exhibited the narrowest host range, infecting only 9 (6.5%) of the 139 *E. coli* isolates tested.

**Figure 2:**
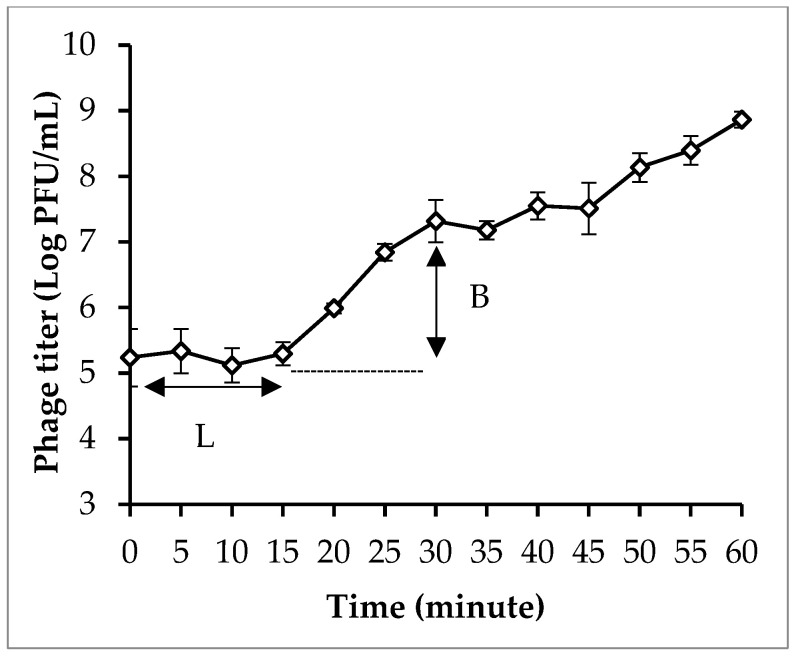
One-step growth curves of phages PEM3 in *E. coli* EM148. L, latent period; B, burst. Error bars show standard deviations.

The one-step growth curve revealed that phage PEM3 had relatively short latent periods of 15 min and a burst size of 223 PFU/cell in *E. coli* EM148 (Figure 2).

**Figure 3:**
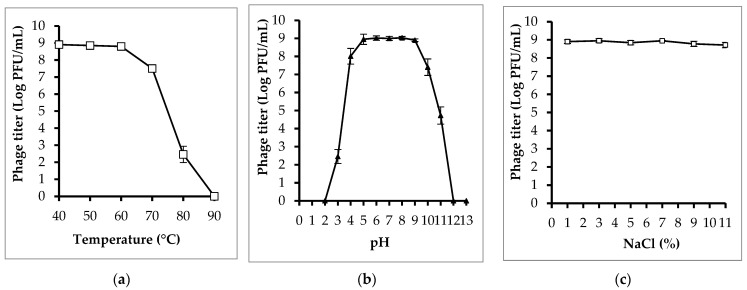
Effects of temperature (**a**), pH (**b**), and NaCl (**c**) on the stability of phage PEM3. Error bars show standard deviations.

The results of the stability test revealed that phage PEM3 had relatively good temperature, pH, and NaCl tolerance (Figure 3). The phage titer remained relatively stable at temperatures ranging from 40 °C to 70 °C, pH values from 4 to 10, and NaCl concentrations from 1% to 11%. At 80 °C, infectious phage particles were still detectable; however, the titer was significantly reduced compared to that at 40 °C to 70 °C. When treated at a temperature of 90 °C for 30 min, the phage was completely inactivated. Similarly, phage particles were found to be not infectious when incubated at pH < 3 and pH > 11 for 60 min.

### 3.5. Efficacy of Phage PEM3 against EM148 in LB Broth and Raw Milk

**Figure 4:**
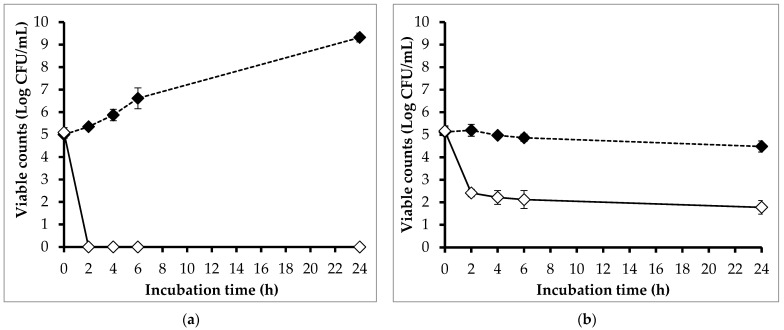
Effect of phage PEM3 on the viability of *E. coli* EM148 in LB broth stored at 24 °C (**a**) and 4 °C (**b**). *E. coli* EM148 was inoculated in 5 mL of LB broth at a final concentration of 10^5^ CFU/mL without (dashed line) and with phage PEM3 at 10^8^ PFU/mL (solid line). Error bars show standard deviations.

Figure 4 shows the effect of phage PEM3 on the viability of EM148 grown in LB broth incubated at 24 °C and 4 °C. At 24 °C, the viable counts of EM148 in the controls increased gradually and reached a plateau at 24 h (Figure 4a). On the contrary, the viable counts of EM148 in the treatments were reduced to below a detectable level (<10 CFU/mL) after 2 h of incubation, and the regrowth of EM148 was not observed at the end of the experiment period.

In the trial at 4 °C, the viable counts of EM148 in the controls were found to be unchanged during 24 h of incubation, whereas those in the phage treatments were significantly decreased (Figure 4b). Compared to the controls, the viable count of EM148 in the treatments was reduced by 2.79 log after 2 h of incubation, and this reduction was maintained throughout the experiment.

**Figure 5:**
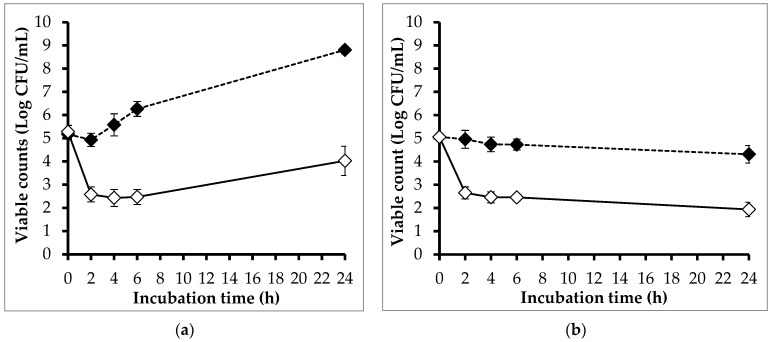
Effect of phage PEM3 on the viability of *E. coli* EM148 in raw milk stored at 24 °C (**a**) and 4 °C (**b**). *E. coli* EM148 was inoculated in 5 mL of raw milk at a final concentration of 10^5^ CFU/mL without (dashed line) and with phage PEM3 at 10^8^ PFU/mL (solid line). Error bars show standard deviations.

The effects of phage PEM3 on the viability of EM148 in raw milk stored at 24 °C and 4 °C were also investigated (Figure 5). In treatments at 24 °C, phage PEM3 lowered the viable counts of EM148 by 2.35, 3.15, and 3.79 log at 2, 4, and 6 h, respectively, compared to the controls (Figure 5a). At the end of experiment (24 h), the regrowth of EM148 occurred in the treatments; however, a 4.78 log reduction of the viable counts was still observed when compared to the controls (Figure 5a).

At 4 °C, the viability of EM148 in the controls remained steady and close to the initial inoculum throughout the experimental period (Figure 5b). In contrast, the viable counts of EM148 in the treatments were significantly decreased by 2.31 and 2.37 log after 2 h and 24 h of incubation, respectively, compared to those in the controls (Figure 5b).

## 4. Discussion

In this study, the prevalence of *E. coli* in raw milk was 34.75%. This result is in agreement with the findings of Liu et al. (2021) in northern China (34.4%) [29]. However, the *E. coli* contamination rate in raw milk in our study was lower than those reported in Malaysia (45%), Pakistan (50%), Egypt (52%), Bangladesh (75%), and India (81.1%) [41,42,43,44,45]. On the contrary, the prevalence of *E. coli* in raw milk found in the present study was higher than that recorded in a study by Awadallah et al. (2016) (22.4%) [46]. In general, our results indicate that the raw milk collected from Hanoi, Vietnam was highly contaminated with *E. coli*. The presence of *E. coli* in raw milk does not only indicate fecal contamination of milk but also reveals improper hygiene and sanitary conditions during milking, irregular washing and sterilization of dairy equipment, and infections of dairy cows that are used for milking purposes [47]. In addition, the high prevalence of *E. coli* in raw milk implies the risk that other enteric pathogens may co-exist [48]. It is worth noting that the differences in *E. coli* contamination rates among various studies may be due to the differences in locality, sample numbers, sampling seasons, isolation procedures, and milk types.

A majority of *E. coli* clones are harmless to humans; however, several strains have acquired virulence factors that enable them to cause human diseases. Pathogenic *E. coli* targeting the human intestine is categorized into six groups, as follows: Shiga-toxin-producing *E. coli* (STEC); enteropathogenic *E. coli* (EPEC); enterotoxigenic *E. coli* (ETEC); enteroaggregative *E. coli* (EAEC); enteroinvasive *E. coli* (EIEC); and diffusely adherent *E. coli* (DAEC) [49,50]. Among these, STEC has been considered as the most important pathogen for the dairy industry since dairy cows are known to harbor STEC in their gastrointestinal tract and excrete the pathogens in their feces [51,52,53]. Also, the teats and udders of animals have been reported to be easily contaminated with feces when cleaning methods and udder hygiene practices are not properly followed, which leads to milk contamination during milking [54]. Another factor making STEC the most important pathogen for the dairy industry is their low dose infection (5–50 cells) compared to those of other *E. coli* groups [53]. STEC contains various serogroups; however, the O157 serogroup and six other non-O157 serogroups (O26, O103, O111, O121, O45, and O145) have been reported as the most common serogroups associated with STEC outbreaks [55]. The ability of STEC to cause serious human diseases is mainly associated with their production capacity of Stx. Based on their antigen differences, the Stx family has been divided into two groups, Stx1 and Stx2. The Stx2 is more clinically important than Stx1, as Stx2 infection has a higher probability of HUS development compared to either Stx1 or both Stx1 and Stx2 [56]. Another crucial virulence factor for STEC to cause human diseases is the *eae* gene encoding the outer membrane adhesin, also called intimin, which allows the pathogens to attach to intestinal epithelial cells. It has been reported that *eae* has frequently been detected in clinical STEC (FAO, 2022). In addition, hemolysin is considered another important virulent factor for STEC. A gene (*ehxA*) encoding plasmid-carried enterohemolysin is usually found in STEC isolates linked to diarrheal disease and HUS [57]. In this study, 3.6% (5/139) of the isolated *E. coli* strains were identified as STEC carrying the *stx1* gene, corresponding to 1.25% (5/400) of the milk samples, indicating the low contamination rate of STEC in milk. The findings in this study align with those of previous studies from Ireland and the USA reporting 0.8–3.2% of raw milk samples contaminated with STEC [58,59]. A recent review of data from a variety of studies also showed that the prevalence of STEC in raw milk samples in Europe ranged from an undetectable level to 5.7% [60]. The occurrence of STEC in raw milk in the current study was lower than in a study in Iran, which found that 6/150 (4%) raw milk and cheese samples tested positive for STEC [61]. A higher prevalence of STEC was also observed in a study conducted in Egypt, where STEC was present in 36/125 (28.8%) raw milk and dairy samples [62]. Recent studies utilized DNA-based methods to detect *stx* genes in raw milk. However, it is important to note that the occurrence of *stx* genes in raw milk does not always indicate the presence of viable cells of STEC, as *stx* genes can be present in non-pathogenic organisms, in phages, or unlinked with bacterium [63]. Therefore, a culturing method is still needed to confirm the presence of viable STEC cells in raw milk samples. Therefore, it is necessary to differentiate between the prevalence of *stx* genes and the prevalence of viable STEC cells. The incidence of *stx* genes in raw milk samples is usually reported to be significantly higher than the prevalence of viable STEC cells [58,59]. In the present study, isolated STEC strains only carried the *stx1* gene, and none of the isolates were positive for *stx2*. This finding is in line with previous studies reporting that *stx1* is the predominant gene in *E. coli* isolates of dairy food origin [64].

The high rate (25.18%) of MDR found in raw milk in this study is reasonable since Vietnam is known to have one of the highest antibiotic resistance rates in Asia. This could be attributed to the significant overuse and misuse of antibiotics in both human and animal health sectors in Vietnam [65,66]. Mastitis, infectious foot, respiratory, reproductive, and diarrhea diseases have been recognized as the most common diseases of dairy cows [67]. Antibiotics, such as penicillin, cephalosporins, and sulfonamides, are often selected to treat these diseases [68]. This could partly explain the high resistance rates to co-trime (27.34%) and ampicillin (27.34%) observed in the present study. Additionally, the *E. coli* isolated in this study were also highly resistant to streptomycin (25.18%). This may be due to the common practice of using streptomycin in combination with penicillin in treating mastitis and other diseases of dairy cows in Vietnam, as they are known for their synergistic effect, low cost, and viability. These results are consistent with those of previous studies reporting that *E. coli* isolated from dairy products exhibited high resistance rates to ampicillin, tetracycline, and co-trime [32,61,69]. Another factor contributing to the high antibiotic resistance rate of *E. coli* isolated from raw milk is the widespread use of antibiotics not only for treating diseases but also for prevention and growth promotion [68]. For example, prophylactic antibiotics through intramammary infusions were applied to 75% of dairy cows in the United States for the prevention of mastitis [70]. The use of antibiotics for disease treatment, prevention, and growth promotion results in residues of antibiotics in milk, consequently leading to the emergence of antibiotic-resistant clones in milk. This is considered the main reason for the presence of MDR in raw milk. However, it cannot be excluded that MDR from bovine feces may directly/indirectly contaminate raw milk during the milking process [50].

The emergence and spread of ESBLEC and colistin-resistant *E. coli* (CREC) has been recognized as a global health concern [71,72]. In our study, seven ESBLEC and four CREC were isolated from raw milk. This is the first report of the co-occurrence of *β*-lactamase encoding genes (*bla_TEM_*, *bla*_CTX-M-1_) and the colistin resistance gene (*mcr-1*) in *E. coli* recovered from raw milk in Vietnam. Similarly, Fillioussis et al. (2020) has reported, for the first time, the isolation of *mcr-1*-postive ESBLEC in raw milk in Greece [73]. A total of 89 *E. coli* strains were isolated from 400 samples, and 6 out of 89 (6.7%) isolates were phenotypically determined as ESBL producers. All six ESBLEC were positive for *mcr-1*, five for *bla_TEM-1_*, three for *bla*_CTX-M_, and three for *bla_SHV_* [73]. Recently, the co-occurrence of ESBL genes and *mcr-1* in *E. coli* isolated from raw milk was also reported in China [74]. A total of 2038 milk samples were used to isolate 249 *E. coli* strains. Among them, five (2%) strains were simultaneously positive for *mcr-1* and ESBL genes [9]. Colistin is not the most common drug for treating diseases in dairy cows, suggesting that the *mcr-1*-positive ESBEC in this study may have originated from bovine feces or the environment, or the use of cephalosporins has resulted in the cross-resistance of cephalosporins and colistin.

Due to growing consumer demand for natural, healthy, and nutritious milk products with a long shelf life, the non-thermal sterilization method capable of controlling pathogens in raw milk is needed to replace pasteurization [75]. Recently, bacteriophages have emerged as promising natural food additives to control foodborne pathogens [76,77,78,79]. In this study, phage PEM3 showed great efficacy in controlling multidrug-resistant *E. coli* in raw milk stored at 4 °C and 24 °C. The application of phage PEM3 resulted in significant reductions in viable counts of the bacterial host, ranging from 2.31 log to 3.79 log CFU/mL. This is the first report demonstrating that phage was effective in killing *mcr-1*-positive ESBLEC in raw milk. The findings are in agreement with previous studies evaluating the effect of phages on the viability of *E. coli* in milk. A study conducted by Mclean et al. (2013) reported that the addition of a phage cocktail containing three phages reduced *E. coli* ATCC 25922 and *E. coli* O127:H6 in raw milk incubated at refrigeration temperature (5–9 °C) and 25 °C to under the detection limit [80]. In another study performed by Grygorcewicz et al. (2020), the application of phage ECPS-6 decreased *E. coli* O157:H7 in raw milk kept at 4 °C and 25 °C by 1.5 log and 4.74 log CFU/mL, respectively [81]. It is noteworthy that the efficacy of phages in reducing the viable cells of bacterial hosts in milk depends on the multiplicity of infection (MOI), the initial inoculum of the bacterial host into milk, the incubation temperature, and the milk properties, thus making it difficult to compare the antibacterial activities of phages across different studies. In our previous studies, several phages against various foodborne pathogens (*E. coli*, *Salmonella*, *Campylobacter*, and *S. aureus*) have been successfully isolated from different food samples (pork, chicken meat, beef, and chicken organs) [17,22,26,40,82,83]. In this study, phage PEM3 was also recovered from food (raw milk), reinforcing the concept that foods are ideal sources for the isolation of phages against foodborne pathogens.

## 5. Conclusions

In summary, raw milk in Vietnam was found to be highly contaminated with *E. coli.* The *E. coli* isolates showed high resistance rates to co-trime, ampicillin, streptomycin, and tetracycline. This is the first report on the presence of multidrug-resistant *E. coli* strains harboring *bla_TEM_*, *bla*_CTX-M-1_ and *mcr-1* gene in raw milk in Vietnam. Phage PEM3 effectively reduced the viable counts of multidrug-resistant *E. coli* isolate (EM148), indicating its potential for controlling MDR-*E. coli* strains in food.

## Figures and Tables

**Table 1:** Primer for detecting virulence-associated genes.

Target Gene	Primer	Primer Sequence (5′–3′)	Amplicon Size (bp)	References
*wzx_O45_*	O45-F	GGGGCTGTCCAGACAGTTCAT	890	[35]
	O45-F	TGTACTGCACCCAATGCACCT		
*wzx_O103_*	O103-F	GCAGAAAATCAAGGTGATTACG	740	
	O103-R	GGTTAAAGCCATGCTCAACG		
*stx1*	stx1-F	TGTCGCATAGTGGAACCTCA	655	[36]
	stx1-R	TGCGCACTGAGAAGAAGAGA		
*wzqE_O121-_wzqF_O121_*	O121-F	TCAGCAGAGTGGAACTAATTTTGT	587	[35]
	O121-R	TGAGCACTAGATGAAAAGTATGGCT		
*wzx_O145_*	O145-F	TCAAGTGTTGGATTAAGAGGGATT	523	
	O145-R	CACTCGCGGACACAGTACC		
*stx2*	stx2-F	CCATGACAACGGACAGCAGTT	477	[36]
	stx2-R	TGTCGCCAGTTATCTGACATTC		
*wzx_O26_*	O26-F	AGGGTGCGAATGCCATATT	417	[35]
	O26-R	GACATAATGACATACCACGAGCA		
*eae*	eae-F	CATTATGGAACGGCAGAGGT	375	[36]
	eae-R	ACGGATATCGAAGCCATTTG		
*rfb_O157_*	O157-F	CAGGTGAAGGTGGAATGGTTGTC	296	[35]
	O157-F	TTAGAATTGAGACCATCCAATAAG		
*wzx_O111_*	O111-F	TGCATCTTCATTATCACACCA	230	[35]
	O111-R	ACCGCAAATGCGATAATAACA		
*ehxA*	ehxA-F	GCGAGCTAAGCAGCTTGAAT	199	[36]
	ehxA-R	CTGGAGGCTGCACTAACTCC		

**Table 2:** Primers for detecting *β-*lactamase encoding genes.

Target Gene	Primer	Primer Sequence (5′–3′)	Amplicon Size (bp)
*bla*_TEM_	TEM-F	GGTCGCCGCATACACTATTCTC	372
	TEM-R	TTTTATCCGCCTCCATCCAGTC	
*bla*_SHV_	SHV-F	CCAGCAGGATCTGGTGGACTAC	231
	SHV-R	CCGGGAAGCGCCCTCCAT	
*bla*_CTX-M-1_	CTX-M1-F	GAATTAGAGCGGGAGTCGGG	588
	CTX-M1-R	CACAACCCAGGAAGCAGGC	
*bla*_CTX-M-2_	CTX-M2-F	GATGGCGACGCTACCCC	107
	CTX-M2-R	CAAGCCGACCTCCCGAAC	
*bla*_CTX-M-9_	CTX-M9-F	GTGCAACGGATGATGTTCGC	475
	CTX-M9-R	GAAACGTCTCATCGCCGATC	
*bla*_CTX-M-8/25_	CTX-M8/25-F	GCGACCCGCGCGATAC	186
	CTX-M8/25-R	TGCCGGTTTTATCCCCG	

**Table 3:** Primers for the detection of *mcr* genes.

Target Gene	Primer	Primer Sequence (5′–3′)	Amplicon Size (bp)	Reference
*mcr-1*	*mcr1* f	AGTCCGTTTGTTCTTGTGGC	320	[38]
	*mcr1* r	AGATCCTTGGTCTCGGCTTG		
*mcr-2*	*mcr2* f	CAAGTGTGTTGGTCGCAGTT	715	[38]
	*mcr2* r	TCTAGCCCGACAAGCATACC		
*mcr-3*	*mcr* 3 f	AAATAAAAATTGTTCCGCTTATG	929	[38]
	*mcr3* r	AATGGAGATCCCCGTTTTT		
*mcr-4*	*mcr4* f	TCACTTTCATCACTGCGTTG	1116	[38]
	*mcr4* r	TTGGTCCATGACTACCAATG		
*mcr-5*	*mcr5* f	ATGCGGTTGTCTGCATTTATC	1644	[39]
	*mcr* 5 r	TCATTGTGGTTGTCCTTTTCTG		

**Table 4:** Antibiotic resistance profile of *E. coli* isolates from raw milk.

Group	Name of Antibiotic	MIC Breakpoint (µg/mL)	Number (%) of Resistant Strains		Total (*n* = 139)
			Ba Vi (*n* = 61)	Gia Lam (*n* = 78)	
Penicillin	ampicillin	32	22 (36.07)	16 (20.51)	38 (27.34)
Cephalosporins	cefotaxime	4	5 (8.2)	2 (2.56)	7 (5.04)
	ceftazidime	16	2 (3.28)	1 (1.28)	3 (2.16)
	cefoxitin	32	1 (1.64)	3 (3.85)	4 (2.88)
	cefepime	16	5 (8.2)	2 (2.56)	7 (5.04)
Carbapenems	meropenem	4	0	0	0
Polymyxins	colistin	4	2 (3.28)	2 (2.56)	4 (2.88)
Tetracyclines	tetracycline	16	21 (34.43)	11 (14.1)	32 (23.02)
Aminoglycosides	streptomycin	32	22 (36.07)	13 (16.67)	35 (25.18)
	gentamicin	16	2 (3.28)	1 (1.28)	3 (2.16)
Macrolides	azithromycin	32	13 (21.31)	0	13 (9.35)
Phenicols	florfenicol	16	6 (9.84)	5 (6.41)	11 (7.91)
Sulfonamide	co-trime	4/76	27 (44.26)	11 (14.1)	38 (27.34)
Fluoroquinolones	ciprofloxacin	4	2 (3.28)	1 (1.28)	3 (2.16)
Quinolones	nalidixic acid	32	3 (4.92)	2 (2.56)	5 (3.6)

**Table 5:** Antibiotic resistance pattern of *E. coli* isolates from raw milk.

No. of Antibiotics	Resistance Phenotype	Number (%) of Resistance Isolates		
		Ba Vi (*n* = 61)	Gia lam (*n* = 78)	Total (*n* = 139)
0	-	21 (34.43)	61 (78.21)	82 (58.99)
1	SXT	4 (6.65)	0	4 (2.88)
	FLO	2 (3.28	0	2 (1.44)
	AMP	1 (1.64)	0	1 (0.72)
	CST	0	1 (1.28)	1 (0.72)
2	AZM-SXT	6 (9.84)	0	6 (4.320
	AMP-STR	3 (4.92)	0	3 (2.16)
	AMP-FOX	0	2 (2.56)	2 (1.44)
	TET-SXT	2 (3.28)	0	2 (1.44)
	STR-SXT	1 (1.64)	0	1 (0.72)
3	AMP-FOX-STR	0	1 (1.28)	1 (0.72)
	AMP-STR-CST	1 (1.64)	0	1 (0.72)
	AMP-STR-TET	1 (1.64)	0	1 (0.72)
	TET-AZM-SXT	1 (1.64)	0	1 (0.72)
4	AMP-STR-TET-SXT	4 (6.65)	5 (6.41)	9 (6.47)
	AMP-STR-FLO-SXT	0	1 (1.28)	1 (0.72)
	STR-TET-CST-SXT	1 (1.64)	0	1 (0.72)
5	AMP-STR-TET-FLO-SXT	2 (3.28)	4 (5.13)	6 (4.32)
	AMP-STR-TET-AZM-SXT	4 (6.65)	0	4 (2.88)
	AMP-CTX-FEP-STR-TET	1 (1.64)	0	1 (0.72)
	AMP-GEN-STR-TET-SXT	0	1 (1.28)	1 (0.72)
	FOX-STR-TET-AZM-SXT	1 (1.64)	0	1 (0.72)
6	AMP-CTX-FEP-CAZ-CIP-NAL	1 (1.64)	1 (1.28)	2 (1.44)
	AMP-CTX-FEP-GEN-TET-SXT	1 (1.64)	0	1 (0.72)
	AMP-GEN-STR-TET-FLO-AZM	1 (1.64)	0	1 (0.72)
7	AMP-CTX-FEP-STR-TET-CIP-NAL	1 (1.64)	0	1 (0.72)
	AMP-CTX-FEP-STR-TET-CST-NAL	0	1 (1.28)	1 (0.72)
8	AMP-CTX-FEP-CAZ-STR-TET-FLO-NAL	1 (1.64)	0	1 (0.72)
Resistant ≥ 1		40 (65.57)	17 (21.79)	57 (41.01)
MDR		21 (34.43)	14 (17.95)	35 (25.18)

AMP, ampicillin; CTX, cefotaxime; CAZ, ceftazidime; FOX, cefoxitin; FEP, cefepime; CST, colistin, TET, tetracycline; STR, streptomycin; GEN, gentamycin; AZM, azithromycin; FLO, florfenicol; SXT, co-trime; CIP, ciprofloxacin; NAL, nalidixic acid.

**Table 6:** Phenotypic and genotypic antibiotic resistance of ESBLEC and colistin-resistant isolates.

Isolate ID	Antimicrobial Resistance Profile	ESBL Screening Test	β-Lactamase Encoding Genes	*mcr* Genes
EM2	AMP-CTX-FEP-GEN-TET-SXT	+	*bla_TEM_*, *bla*_CTX-M-1_	ND
EM29	AMP-CTX-FEP-CAZ-CIP-NAL	+	*bla_TEM_*	ND
EM32	STR-TET-CST-SXT	ND	ND	*mcr-1*
EM40	AMP-STR-CST	ND	ND	*mcr-1*
EM42	AMP-CTX-FEP-STR-TET-CIP-NAL	+	*bla_TEM_*, *bla*_CTX-M-1_	ND
EM72	AMP-CTX-FEP-CAZ-STR-TET-FLO-NAL	+	*bla_TEM_*, *bla*_CTX-M-1_	ND
EM75	AMP-CTX-FEP-STR-TET	+	*bla*_CTX-M-1_	ND
EM102	AMP-CTX-FEP-CAZ-CIP-NAL	+	*bla_TEM_*	ND
EM144	CST	ND	ND	*mcr-1*
EM148	AMP-CTX-FEP-STR-TET-CST-NAL	+	*bla_TEM_*, *bla*_CTX-M-1_	*mcr-1*

ND: Not determined. AMP, ampicillin; CTX, cefotaxime; CAZ, ceftazidime; FEP, cefepime; CST, colistin, TET, tetracycline; STR, streptomycin; GEN, gentamycin; FLO, florfenicol; SXT, co-trime; CIP, ciprofloxacin; NAL, nalidixic acid.

## Data Availability

The data that support the findings of this study are available from the corresponding author upon reasonable request.
